# Probabilistic Estimation of Fatigue Strength for Axial and Bending Loading in High-Cycle Fatigue

**DOI:** 10.3390/ma13051148

**Published:** 2020-03-05

**Authors:** Tomasz Tomaszewski, Przemysław Strzelecki, Adam Mazurkiewicz, Janusz Musiał

**Affiliations:** Faculty of Mechanical Engineering, University of Science and Technology, al. Prof. S. Kaliskiego 7, 85-796 Bydgoszcz, Poland; p.strzelecki@utp.edu.pl (P.S.); adam.mazurkiewicz@utp.edu.pl (A.M.); janusz.musial@utp.edu.pl (J.M.)

**Keywords:** Weibull distribution, axial loading, rotary bending, high-cycle fatigue

## Abstract

In this paper, the sensitivity to the type of loads (axial and bending loading) of selected construction materials (AW6063 T6 aluminum alloy, S355J2+C structural steel, and 1.4301 acid-resistant steel) in high-cycle fatigue was verified. The obtained *S-N* fatigue characteristics were described by a probabilistic model of the 3-parameters Weibull cumulative distribution function. The main area of research concerned the correct implementation of the weakest link theory model. The theory is based on a highly-stressed surface area and a highly-stressed volume in the region of the highest stresses. For this purpose, an analytical model and a numerical model based on the finite element method were used. The model that gives the lowest error implemented in specific test conditions was determined on the basis of high-cycle fatigue analysis. For the analyzed materials, it was a highly-stressed volume model based on the weakest link theory.

## 1. Introduction

The fatigue strength/fatigue life must be determined while designing new components for machines subject to loads variable in time. The fatigue characteristic (stress—life *S-N*, strain—life *ε-N*) for the material (unnotched specimens) is multiplied by appropriate coefficients (e.g., cyclic strength, surface finishing, size, shape, cycle asymmetry, and ductility coefficients) to determine the characteristic for a structural component [1,2,3,4]. The procedure for determining the fatigue properties is carried out from testing to analytical and/or numerical models [5,6]. It is a continuously growing field, even in the case of experimental tests [7,8].

The tests are conducted on testing machines, because the specimens are subject to rotary bending or tension/compression loading. The tests under rotary bending loading yield higher loading frequencies (up to 200 Hz) in comparison to axial loading where a standard testing machine reaches up to 20 Hz (for example in [9] 10 Hz was applied). Rotary bending testing machines are less expensive due to their simple design. Rotary bending loading also requires lower loading forces. At 200 MPa, under rotary bending loading with a 46 mm arm and a 5 mm dia. specimen, 578 N is required, whereas in the case of a tensile test, the required force amounts to 2454 N. In both loading cases, normal stress occurs in the specimen, and the difference lies in the loading gradient. The fatigue life determined experimentally was compared in [10]. The fatigue limit for axial loading was 75% to 87% lower than for rotary bending loading.

One of the most commonly used methods to estimate the fatigue limit is the Weibull weakest link theory [11]. Main assumption of the theory is the occurrence of material defects (unevenness, inclusions, cracks, defects) statistically distributed per unit volume. Cracks are initiated in specific units with the most dangerous defects or the weakest links. The propagation of fatigue cracks is independent in different areas. An analyzed area has a finite number of links. Any damage to the links results in damage to the entire component. The size of the defects is smaller than the distance between them. The theory was generalized to the fatigue limit by Kugnel in [12]. In the theory, the fatigue strength is characterized by the Weibull probability distribution defined in [13]. The distribution is characterized by three parameters: a scale parameter *β*, a location parameter *ξ*, and a shape parameter *α*. The shape parameter *α* and the location parameter *ξ* are used as a material constant used to differentiate the ratio of fatigue strength for rotary bending and axial loading.

The effects of non-uniform stress distribution on material fatigue properties is determined using a surface area method (highly-stressed surface area *A_n_*_%_) or a volume method (highly-stressed volume *V_n_*_%_). In this region, the probability of cracking initiation or propagation of an existing defect is higher. *A_n_*_%_/*V_n_*_%_ is defined as the ratio of surface area to volume of material subject to at least *n*% of the maximum stress. Calculation of these parameters is carried out using the finite element method [6,7]. The stress distribution map determined numerically for the analyzed load provides information on the number of highly stressed finite elements.

Analytical methods can be found in literature, which are based on estimation of the ration of the fatigue strengths for the axial and bending stress to calculate the fatigue strength/life for not tested type of loading *φ*. Such models can be found in [14,15,16]. A comparison of these models is presented in [17]. It should be mentioned that the estimated values of *φ* by these models are less than the results estimated in experiments. A 50% probability characteristic was estimated by using such models, but experimental results were different for the scatter band of fatigue life for axial and rotary bending load, which was presented in [18]. This is why authors decide to implement a new approach of the weakest link theory, which changes the fatigue strength and the scatter band of the fatigue life.

In the present paper, a model based on the weakest link theory (surface area and volume approach) was verified. The analyses were carried out for the results of the authors’ own experimental test results for selected materials (S355J2+C structural steel, 1.4301 acid-resistant steel, and AW6063 T6 aluminum alloy). High-cycle fatigue tests were performed under axial and bending loading conditions. The aim of the paper was to determine the results of an analytical model similar to experimental test results.

## 2. Materials and Methods

### 2.1. Analytical Model

**Figure 1:**
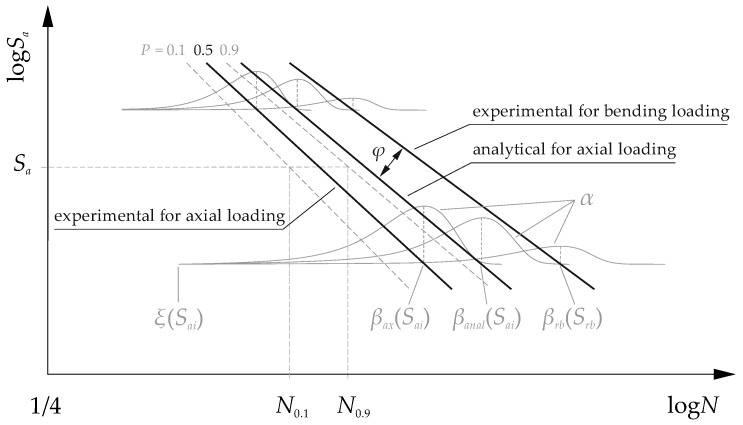
Scheme for *S-N* characteristics estimation taking into account the probability of failure *P* and the corresponding fatigue life *N*_0.1_, *N*_0.9_ for constant stress amplitude *S_a_*.

Due to the different ratio of volume under load and the number of fatigue cracking initiation and propagation points, the fatigue strength for rotary bending loading is higher than for axial loading (tension/compression). Fatigue tests for rotary bending are shorter and more cost-efficient. *S-N* characteristics for rotary bending were used as a basis for the analysis. The fatigue limit for axial loading was estimated on the basis of those results. The probability of failure for any stress level (*P* = 0.1; 0.5; 0.9) was used to extend the relationship between the stress and the number of cycles. The spread of fatigue strength in the high cycle range decreased with increasing stress amplitude, which did not describe the Basquin model with normal distribution presented in the normative document [19]. The Weibull distribution function was used to describe this spread. A graphic representation of the calculations and the Weibull distribution function parameters is pictured in Figure 1. A correction coefficient *φ* was used to evaluate the effects of the loading mode:

(1) $$\varphi =\frac{S_{ax}}{S_{rb}},$$

where *S_ax_* is the fatigue strength for axial loading and *S_rb_* is the fatigue strength for bending loading.

The weakest link theory presented by Weibull in [11] assuming that stressed volume is divided in a finite number of infinitesimal links. The probability of failure in each link is the same. This probability of failure can be represented by the 3-parameters Weibull cumulative distribution function for general volume, and can be expressed by the following [11]:

(2) $$F_{V}=1-\exp\left[-\frac{1}{V_{0}}\;\int_{V}{\left(\frac{N-\xi }{\sigma }\right)}^{\alpha }dV\right],$$

where *V*_0_ is the reference volume, *V* is the volume of material that is subjected to stress, *N* is the fatigue life, *ξ* is the location parameter, *σ* is the scale coefficient, and *α* is the shape parameter in the *S-N* characteristic equation. Equations (3)–(8) kept the same description of the variables.

For uniform stress distribution (subscript 0) (equation axial loading for a round specimen):

(3) $$F_{V_{0}}=1-\exp\left[-\;{\left(\frac{N_{0}-\xi_{0}}{\sigma_{0}}\right)}^{\alpha_{0}}\right].$$

For nonuniform stress distribution (subscript 1), it can be written [20,21]:

(4) $$F{\;}_{V_{1}}=1-\exp\left[-\frac{V_{1}}{V_{0}}\;{\left(\frac{N_{1}-\xi_{1}}{\sigma_{1}}\right)}^{\alpha_{1}}\right].$$

Assuming that shape and location parameters are in common and only differ in the scale parameter for two stress distributions, which is the variable allowing the following to be written:

(5) $$F{\;}_{V_{1}}\;=\;1-\exp\left[-\frac{V_{1}}{V_{0}}\;{\left(\frac{N_{1}-\xi_{0}}{\sigma_{1}}\right)}^{\alpha_{0}}\right].$$

To satisfy the assumption that the probability of failure is the same for two different stress distributions, we have:

(6) $$1-\exp\left[-\;{\left(\frac{N_{0}-\xi_{0}}{\sigma_{0}}\right)}^{\alpha }\right]=1-\exp\left[-\frac{V_{1}}{V_{0}}\;{\left(\frac{N_{1}-\xi_{0}}{\sigma_{1}}\right)}^{\alpha }\right].$$

From Equation (6), the scale parameter for nonuniform stress distribution and uniform stress distribution at the same probability of failure satisfy:

(7) $$\frac{N_{0}-\xi_{0}}{\sigma_{0}}=\frac{N_{1}-\xi_{0}}{\sigma_{1}\cdot {\left(\frac{V_{1}}{V_{0}}\right)}^{\frac{1}{\alpha }}}.$$

It is assumed that the fatigue life is the same (*N*_1_ = *N*_0_), because the fatigue strength changes for the different types of loading. Then, Equation (6) takes the following form:

(8) $$\sigma_{0}=\sigma_{1}\cdot {\left(\frac{V_{1}}{V_{0}}\right)}^{\frac{1}{\alpha }}.$$

Assuming that the Weibull distribution presented above describes an *S-N* characteristic, the location parameter and the scale parameter by regression line can be defined. Finally, the equation for *S-N* characteristics is defined by the following formula:

(9) $$F\left(N\right)=1-\exp\left[-{\left(\frac{\left(N_{i}\right)-\xi \left(S_{ai}\right)}{\beta \left(S_{ai}\right)}\right)}^{\alpha }\right],$$

where *α* is the shape parameter, *N_i_* is the number of cycles, *ξ*(*^S^_ai_*) is the location parameter (*ξ*(*^S^_ai_*) = 10*^n^*^⋅log(*S*^*_ai_*^)+*d*^), *β*(*^S^_ai_*) is the scale parameter (*β*(*^S^_ai_*) = 10*^m^*^⋅log(*S*^*_ai_*^)+*b*^), *d* and *b* are the constant term in the *S-N* characteristics equation, *m* and *n* is the slope coefficient in the *S-N* characteristics equation, subscript *i* is the stress level ordinal number. Equations (10)–(13), (15)–(17) kept the same description of the variables. Substituting the scale parameter to Equation (8) gives:

(10) $$10^{a\cdot \log\left(S_{ai}\right)+b_{0}}\;=10^{a\cdot \log\left(S_{ai}\right)+b_{1}}\cdot {\left(\frac{V_{1}}{V_{0}}\right)}^{\frac{1}{\alpha }}.$$

In Equation (10), it is assumed that the scale coefficient in the *S-N* characteristic for different type loading is the same, which is proven in the following part of this article. The final form of Equation (10) is:

(11) $$b_{0}=b_{1}+\log\left[{\left(\frac{V_{1}}{V_{0}}\right)}^{\frac{1}{\alpha }}\right].$$

The weakest link theory model was used to estimate the fatigue strength for axial load. The equation is presented in [21,22] for axial loading (subscript *ax*):

(12) $$F_{ax}\left(N\right)=1-\exp\left[-{\left(\frac{\left(N_{i}\right)-\xi \left(S_{ai}\right)}{\beta_{ax}\left(S_{ai}\right)}\right)}^{\alpha }\right].$$

For bending loading (subscript *rb*):

(13) $$F_{rb}(N)=1-\exp\left[-\frac{V_{rb}}{V_{ax}}\;{\left(\frac{\left(N_{i}\right)-\xi \left(S_{ai}\right)}{\beta_{rb}\left(S_{ai}\right)}\right)}^{\alpha }\right].$$

**Figure 2:**
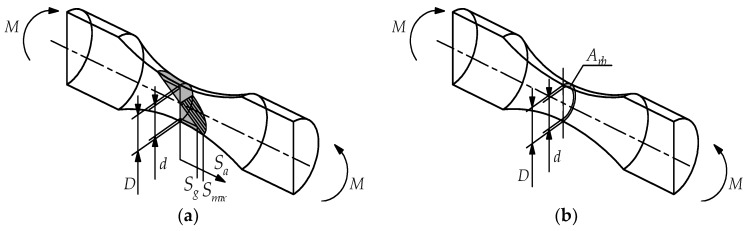
Bending specimen: (**a**) stress distribution, (**b**) surface area *A_rb_*.

Implementation of the Weibull weakest link theory assumes comparison of a highly-stressed volume or a highly-stressed surface area. The surface area was analytically calculated using the following formula:

(14) $$A_{rb}=\frac{{\pi D}^{2}}{4}-\frac{{\pi d}^{2}}{4},\;A_{ax}=\frac{{\pi D}^{2}}{4},$$

where *d* corresponds to *S_g_* = 0.95 *S_max_* (Figure 2).

The constant term *b_ax_* in the *S-N* characteristics equation for axial loading depends on the type of load. For an area, it is defined by the following formula:

(15) $$b_{\mathrm{ax}\;A}=b_{\mathrm{rb}}+\log\left[{\left(\frac{A_{rb}}{A_{\mathrm{ax}}}\right)}^{\frac{1}{\alpha }}\right].$$

The highly-stressed volume was numerically calculated using the finite element method (Section 2.2) for rotary bending (*V_rb V_*) and pure bending (*V_rb S_*). The constant term *b_ax_* for rotary bending takes the form:

(16) $$b_{\mathrm{ax}\;V}=b_{\mathrm{rb}}+\log\left[{\left(\frac{V_{rb\;V}\;}{V_{\mathrm{ax}}}\right)}^{\frac{1}{\alpha }}\right],$$

and for pure bending:

(17) $$b_{\mathrm{ax}\;S}=b_{\mathrm{rb}}+\log\left[{\left(\frac{V_{rb\;S}}{V_{\mathrm{ax}}}\right)}^{\frac{1}{\alpha }}\right].$$

### 2.2. Numerical Model

**Figure 3:**
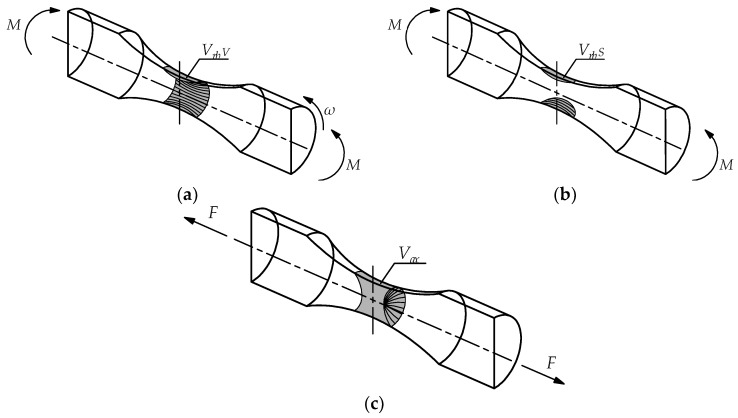
Boundary conditions for round specimen under: (**a**) rotary bending (highly-stressed volume), (**b**) pure bending (highly-stressed volume static), (**c**) axial loading.

Using the weakest link theory model for the highly-stressed volume required the specimen volume to be determined at which the stress exceeds 0.95 *S_max_*. Calculations were carried out using the finite element method software ANSYS Workbench 2020 R1. Figure 3 presents the boundary conditions with highly-stressed volume for rotary bending (*V_rb V_*), pure bending (*V_rb S_*), and axial loading (*V_ax_*).

The geometric model was divided into nearly 1500 elements. Twenty nodal solid elements with three degrees of freedom per node with quadratic displacement behavior (SOLID186) were used. The analyses of the discretization error showed that the satisfactory accuracy of the obtained results is for an average element size of 1 mm. In each of the analyzed materials, the load range was below the yield point. The deformation of the material was in the elastic range. For the tested smooth sample, local stress concentration (including plastic deformation) was not observed. Therefore, it was decided to use the isotropic elastic model (steel: *E* = 2.1 × 10^5^ MPa, *ν* = 0.33; aluminum alloy: *E* = 0.7 × 10^5^ MPa, *ν* = 0.33; where *E* is the modulus of elasticity, *ν* is Poisson’s ratio). Similar linearly elastic analyses were performed in paper [23]. The stress distribution map was used to calculate the highly-stressed volume. The calculations were performed in the module of structural analysis with steady loading and response conditions. The geometry corresponded to the actual dimensions of the specimens.

### 2.3. Experimental Tests

The analytical models were verified on the basis of experimental data for AW 6063 T6 aluminum alloy, S355J2+C structural steel, and 1.4301 acid-resistant steel. The mechanical properties were determined on the basis of monotonic tensile tests according to PN-EN ISO 6892-1:2016 [24]. Table 1 presents the average values.

**Figure 4:**
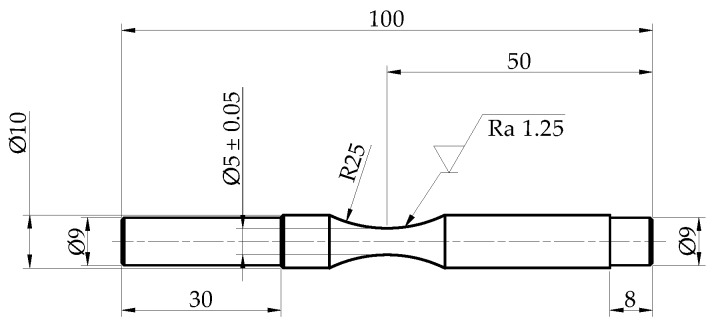
Geometry of the round specimen for fatigue testing.

High-cycle fatigue tests were conducted on a specimen made from a 10 mm drawn bar. The minimum diameter of the working section of the round specimen with a cross-section was 5 mm (Figure 4).

Experimental tests were carried out at axial load on an Instron 8874 hydraulic testing machine and at rotary bending load on the test stand. A symmetric sinusoidal cycle (*R* = −1) was used for the axial load. The tests were carried out in accordance with international standards [19,25,26]. The purpose was to determine the *S-N* characteristic for a load-controlled test. The end of the test criterion was a micro-fracture of the specimen. Figure 7 show a graphic representation of the results. The maximum likelihood method was used to estimate the parameters for a 3-parameters Weibull distribution and was described in [27,28]. Linear regression equation parameters for both loading modes are included in Table 2. A statistical parallelism test was conducted for the characteristics in accordance with [29]. It can be assumed that the characteristics for AW 6063 aluminum alloy are parallel. At this stage, the assumption for the analytical model to estimate the fatigue strength should allow for the parallelism of the characteristics, depending on the material.

Correction coefficient *φ* values were determined on the basis of the obtained data. Table 3 presents the experimental test results. The calculations were conducted for the extreme values of the fatigue strength for a specific range of tests. Structural steel is more sensitive to changes in loading mode.

The analysis of coefficient *a* of the regression line equation involved comparison of the experimental values of gamma distribution. The distributions were determined on the basis of the analysis of the experimental data for the *S-N* characteristics. The static distribution equation was expressed as follows [30,31]:

(18) $$f\left(m\right)=\lambda \cdot \frac{{\left(\lambda \cdot m\right)}^{k-1}}{\Gamma \left(k\right)}\cdot e^{-\lambda \cdot m},$$

where *λ*, *k* are the gamma distribution parameters (size, shape), and *Γ*() is the Euler function.

Table 4 shows the gamma distribution parameters for coefficient *a*. The fifth and sixth columns indicate the results of the statistical test *χ*^2^ in accordance with the following formula [31]:

(19) $$\chi^{2}=\sum_{i=1}^{r}\frac{{\left(n_{i}-np_{i}\right)}^{2}}{np_{i}},$$

where *r* is the number of classes, *n_i_* is the empirical numbers of subsequent classes, *p_i_* is the theoretical frequencies of the class and *n* is the sample size. 

**Figure 5:**
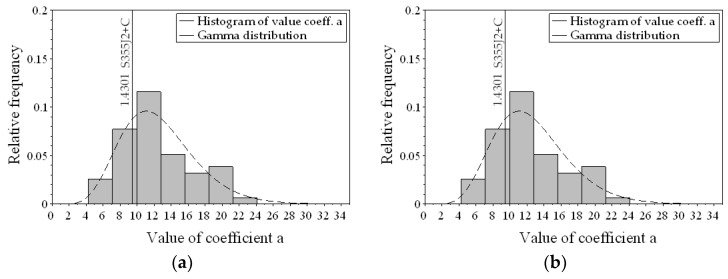
Distribution of coefficient *a* for steel, smooth specimen: (**a**) bending loading, (**b**) axial loading.

The gamma distribution values were similar for the distribution of coefficient *a* in the tensile and bending tests, which may indicate a similar distribution. To verify the null hypothesis on the uniform distribution in both loading cases, a conformity test for two populations was conducted, the Mann–Whitney U test [29]. Value of *W* amounted to 1011.5, and the *p*-value amounted to 0.8646 (it was assumed that the null hypothesis can be rejected if the *p*-value is lower than 0.05). This fact means that there is no basis to reject the null hypothesis. Figure 5 shows the distribution of coefficient *a*, taking into account the values obtained experimentally for the steel materials.

## 3. Results

Verification of the results included estimation of the fatigue strength on the basis of analytically determined coefficients *φ*. The *S-N* characteristics for the axial loading were calculated in accordance with the data regarding the rotary bending loading. The calculations were carried out in accordance with Figure 1. The analyzed range of the fatigue life corresponded to the range of the experimental tests.

**Figure 6:**
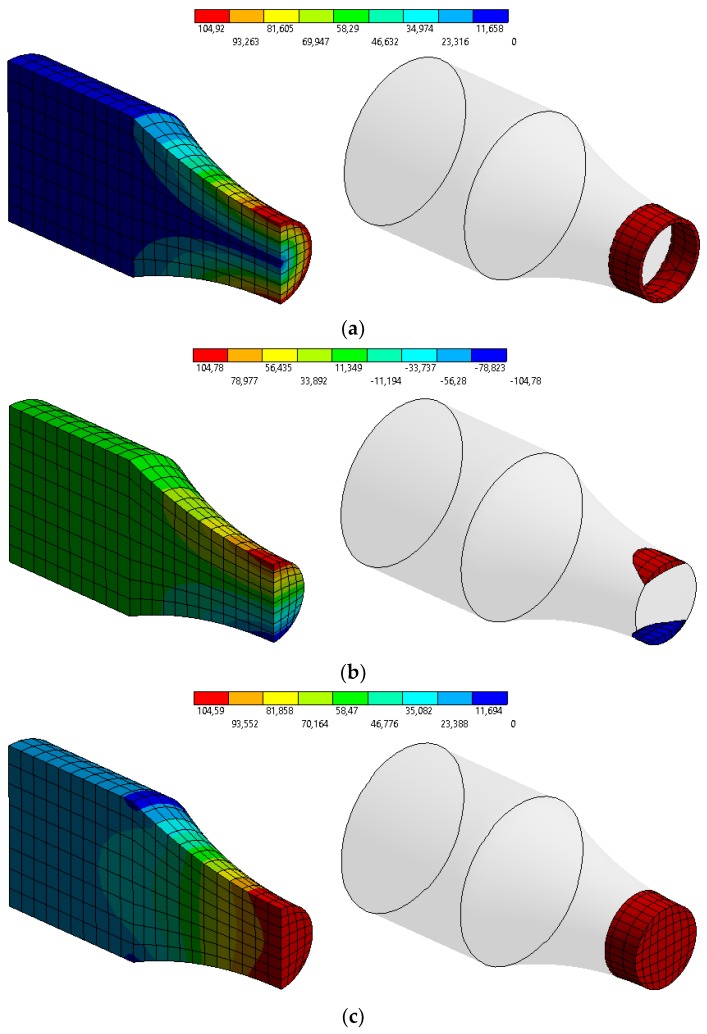
Normal stress distribution map (nominal stress 100 MPa) for round specimen under: (**a**) rotary bending (highly-stressed volume), (**b**) pure bending (highly-stressed volume static), (**c**) axial loading.

Highly-stressed volume calculations were carried out using the finite element method. Figure 6 shows the boundary and a normal stress distribution map for the analyzed loading. The stress and volume for specific elements were stored in the text file processed in R software, ver. 3.5.1 [32]. Elements with stress exceeding 0.95 *S_max_* were determined. Furthermore, the volumes of the determined elements were summed up. An identical procedure was used for three loading cases. Coefficient *b_ax_* was calculated according to Equations (16) and (17). The other parameters of the Weibull distribution were taken from rotary bending. The results of the analysis were included in Table 5, Table 6 and Table 7.

**Figure 7:**
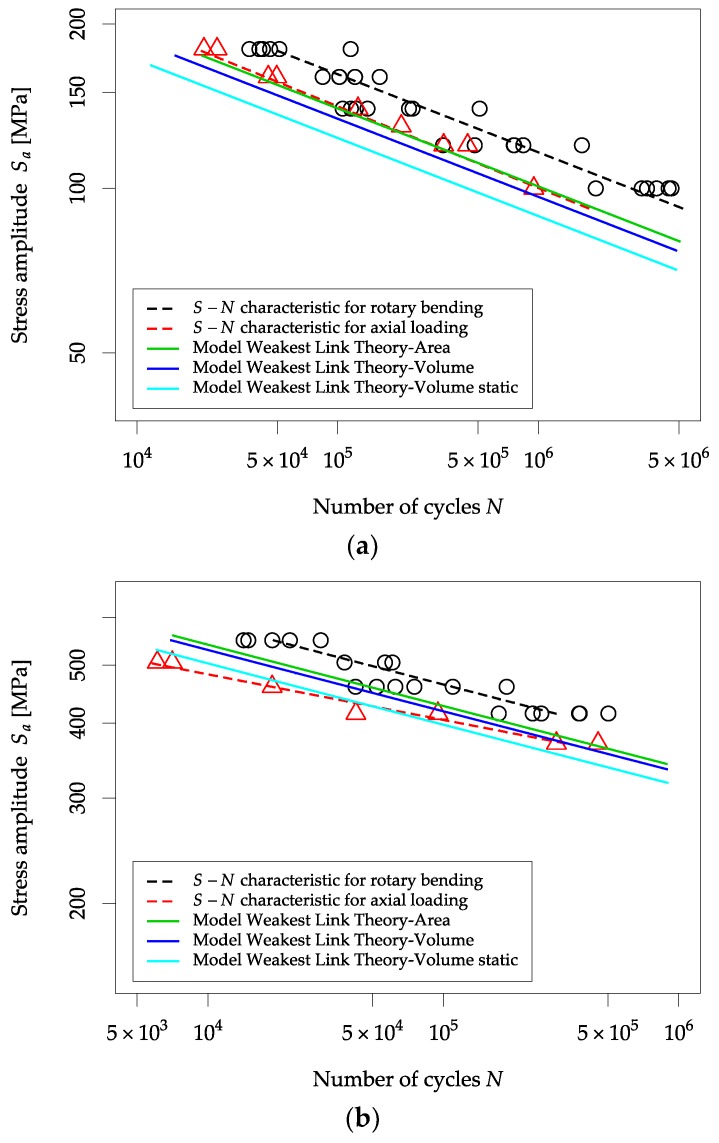

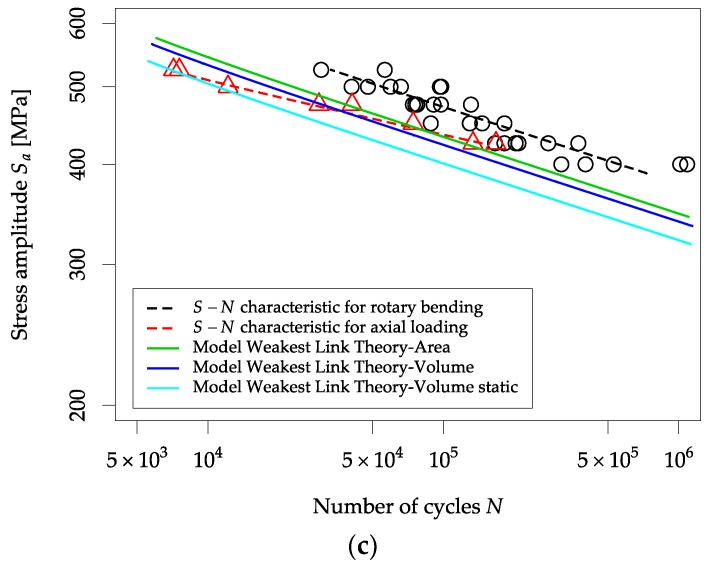
Fatigue characteristic determined based on experimental tests and analytical models for: (**a**) AW 6063 T6, (**b**) S355J2+C, (**c**) 1.4301.

Figure 7 presents the results of the estimated fatigue characteristics for the Weibull model with different defined highly-stressed regions.

For all materials, the model with the highly-stressed volume static gave the lowest fatigue strength, so it is a conservative approach. However, the highly-stressed area model gave the highest fatigue strengths. The estimated fatigue characteristics are on the safe side, i.e., the analytical fatigue strength is lower than the experimental one. If the highly-stressed volume decreases, the fatigue strength also decreases. The highly-stressed area model gives the highest fatigue strengths. By using the highly-stressed volume model, it gets results between the two other models, but it is closer to the highly-stressed area model.

The results were analyzed to fit the analytical fatigue characteristics to the experimental characteristics with a 50% probability of failure. For the AW6063 T6 aluminum alloy, the best fit is for the highly-stressed area model, for the S355J2+C structural steel, it is the highly-stressed volume model and for the 1.4301 acid-resistant steel, it is the highly-stressed volume static model. The indicated discrepancies result from the different structure of the analyzed materials and the mechanisms accompanying the initiation and fatigue crack growth. This shows the correctness of the selected materials because differences in sensitivity to the type of load are noticeable in the results and models used. In this case, the procedure for choosing the model for estimating fatigue properties is dependent on the type of material being analyzed.

As was assumed in Section 2.1, all estimated characteristics are parallel. Only for the AW6063 T6 aluminum alloy, the experimental fatigue characteristics are parallel, but the estimated fatigue strength by the Weibull model is very close to the experimental results.

The characteristics include experimental points approximated to a 50% probability of failure. The quantitative evaluation of the correspondence of empirical and theoretical data (based on the analytical model) was carried out based on the standard deviation of the residual *S_e_* and residual variance *V_e_* (Table 8 and Table 9). The standard deviation and variance for the analytical methods are in bold, which are lower than that calculated from the experimental data for rotary bending loading. In those cases, the results obtained using a specific analytical method are more accurate and can be found within the scatter range of the experimental data.

**Figure 8:**
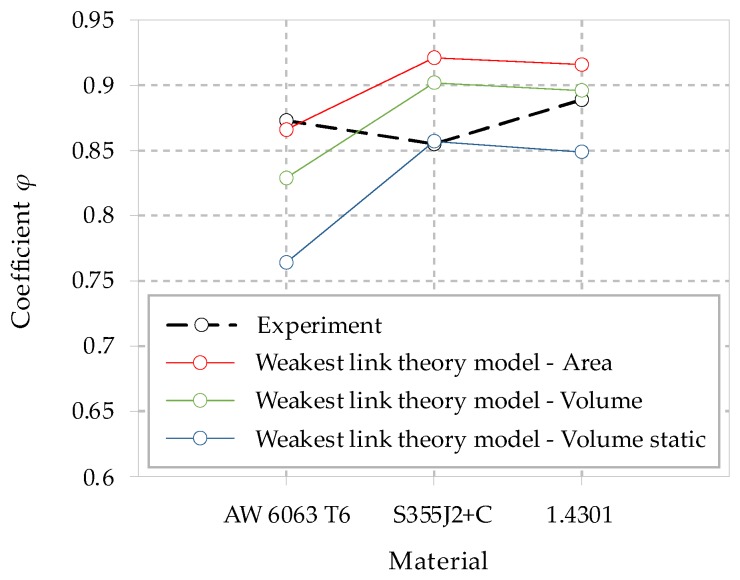
Values of the coefficient *φ*.

Figure 8 presents the correction coefficient *φ* values determined experimentally and analytically for various models of the weakest link theory. The figure shows that the experimental values of the coefficient *φ* are in a narrow range of values (0.86–0.89).

## 4. Discussion and Conclusions

The *S-N* characteristics were determined experimentally for axial loading and rotary bending loading. For the axial loading, the slope of the linear regression *a* is similar to the arithmetical average for the histogram. For rotary bending loading, values close to the modal value were obtained. Thus, the *S-N* characteristics are similar to the test results presented by other researchers, which can be found in e.g., [33] and summaries in [34], where it is mentioned that the coefficient *a* is between 5–20 and generally 10.

The correction coefficient *φ* for the experimental data is within 0.86–0.89, corresponding to the general sensitivity of the materials to the loading mode, the proposed value can be found in e.g., [35]. An initial evaluation of the correctness of the implementation of the analyzed models was conducted on the basis of a comparison of the coefficient *φ* determined experimentally and analytically. It should be mentioned that the correction coefficient *φ* does not have a constant value. It is change for different materials. This can be seen in Figure 8.

The analysis involved verification of the weakest link theory from the surface area and volume perspective. Calculations were carried out using a probabilistic model for the *S-N* characteristics on the basis of the Weibull distribution. Conclusions were proposed for the results of the 50% probability of failure. The best estimation results were obtained for the highly-stressed volume method. For AW6063 T6 aluminum alloy, the surface area weakest link theory method yielded the lowest error compared to the volume method. This probably occurred, because the slope coefficient *a* for the bending and axial loading are very close, see Table 2. In this method, correlating the *φ* to the shape coefficient *α* from the Weibull distribution provides a change in value similar to the observed experimental data (Figure 8). It can be concluded that the closer the value of the coefficient *φ* estimated by analytical method to the experimental result, the less the error of predicted fatigue strength, which is seen by comparison in Figure 8 and Table 9. The rationale for the use of the weakest link theory is also supported by the fact that it is commonly used to determine the effect of element size on fatigue strength [21,36,37,38]. Additionally, by using the Weibull distribution, the fatigue characteristic for any probability can be estimated, which is not recommended for a normal distribution. This statement can be found in [39].

A proposed estimation of the parameters of the Weibull distribution for a different type of loading can be easily adapted to determine the fatigue strength for defining the fatigue life (“fatigue limit”). For this purpose, the scale and location parameter should be a substitute as a constant value of stress amplitude. Then, the distribution will describe the fatigue strength. To adopt the proposed method to low cycle fatigue, it must be submitted to the Basquin equation in the scale and location parameter into the Mason-Coffin equation. The method presented in this article is a universal method because it can be adapted to other fatigue life/strength calculations.

## Figures and Tables

**Table 1:** Mechanical properties for selected materials.

Material	Modulus of Elasticity	Tensile Strength	Yield Strength	Reduction of Area	Elongation
	*E,* MPa	*R_m_*, MPa	*R_e_*, MPa	*Z*, %	*A*, %
AW 6063 T6	69,556	243	201	75.5	10.5
S355J2+C	206,060	806	678	12	65.1
1.4301	206,553	803	561	78.3	43.8

**Table 2:** Fatigue characteristic *S-N* parameters.

Material	Type of Loading	Regression Line Described by Equation (9)				
		*α*	*a*	*b*	*n*	*d*
AW 6063 T6	Axial	2.31	−6.69	19.45	−0.25	1.02
	Bending	2.31	−6.99	20.51	−0.25	1.02
S355J2+C	Axial	2.87	−13.21	39.50	−0.64	3.88
	Bending	2.87	−9.82	31.24	−0.64	3.88
1.4301	Axial	2.58	−15.32	45.49	−0.74	5.06
	Bending	2.58	−10.48	33.07	−0.74	5.06

**Table 3:** Coefficient *φ*.

Material	*φ*
AW 6063 T6	0.873
S355J2+C	0.855
1.4301	0.889

**Table 4:** Values of gamma distribution parameters for the coefficient *a*.

Type of Load	Parameter *λ*	Parameter *k*	Number of Materials	Statistics Test *χ*^2^	Critical Value of Stat. *χ*^2^*_kr_*	Arithmetic Average	Median	Dominant
Axial loading	0.667	8.501	36	0.71	7.815	12.7	12.15	9.5
Bending loading	0.654	8.257	55	0.15	7.815	12.6	11.8	11.9

**Table 5:** Values of the 3-parameters Weibull models for AW 6063 T6.

Data	AW 6063 T6				
	*α*	*a*	*b*	*n*	*d*
Weakest link theory model—Area	2.31	−6.99	20.08	−0.25	1.02
Weakest link theory model—Volume	2.31	−6.99	19.95	−0.25	1.02
Weakest link theory model—Volume static	2.31	−6.99	19.70	−0.25	1.02

**Table 6:** Values of the 3-parameters Weibull models for S355J2+C.

Data	S355J2+C				
	*α*	*a*	*b*	*n*	*d*
Weakest link theory model—Area	2.87	−9.82	30.89	−0.64	3.88
Weakest link theory model—Volume	2.87	−9.82	30.80	−0.64	3.88
Weakest link theory model—Volume static	2.87	−9.82	30.58	−0.64	3.88

**Table 7:** Values of the 3-parameters Weibull models for 1.4301.

Data	1.4301				
	*α*	*a*	*b*	*n*	*d*
Weakest link theory model—Area	2.58	−10.48	32.67	−0.74	5.06
Weakest link theory model—Volume	2.58	−10.48	32.57	−0.74	5.06
Weakest link theory model—Volume static	2.58	−10.48	32.33	−0.74	5.06

**Table 8:** Values of the standard error and the coefficient of variation for the experimental results.

Data	AW 6063 T6		S355J2+C		1.4301	
	*S_e_*	*V_e_*	*S_e_*	*V_e_*	*S_e_*	*V_e_*
Experiment—axial loading	4.3	3.0	10.5	2.4	7.5	1.6
Experiment—bending loading	9.7	6.7	21.3	4.9	31.6	7.2

**Table 9:** Values of standard error of the estimate and the coefficient of variation for the analytical models.

Data	AW 6063 T6		S355J2+C		1.4301	
	*S_e_*	*V_e_*	*S_e_*	*V_e_*	*S_e_*	*V_e_*
Weakest link theory model—Area	**5.8**	**4.1**	49.7	11.4	26.8	5.6
Weakest link theory model—Volume	12.3	8.6	40.0	9.2	**21.6**	**4.5**
Weakest link theory model—Volume static	24.1	16.8	**23.2**	**5.3**	33.0	6.9

## References

[1] Hobbacher A.F. (2008). IIW Document IIW-1823-07 Recommendations for Fatigue Design of Welded Joints and Components.

[2] Kocańda S., Szala J. (1997). Basis of Calculation of Fatigue.

[3] Kocak M., Webster S., Janosch J.J., Ainsworth R.A., Koers R. (2006). FITNET Fitness-For-Service PROCEDURE—FINAL DRAFT MK7.

[4] Kurek A., Kurek M., Łagoda T. (2019). STRESS-LIFE CURVE FOR HIGH AND LOW CYCLE FATIGUE Andrzej Kurek, Marta Kurek, Tadeusz Łagoda. J. Theor. Appl. Mech..

[5] Ai Y., Zhu S.P., Liao D., Correia J.A.F.O., De Jesus A.M.P., Keshtegar B. (2019). Probabilistic modelling of notch fatigue and size effect of components using highly stressed volume approach. Int. J. Fatigue.

[6] Fröschl J., Decker M., Eichlseder W. (2011). Neuer Ansatz zur Bewertung von Stützwirkung und statistischem Größeneinfluss im Auslegungsprozess. Mater. Test..

[7] Pereira A.B., Fernandes F.A.O., de Morais A.B., Carvalhoso P. (2019). Development of a delamination fatigue testing machine for composite materials. Machines.

[8] Bathias C. (2006). Piezoelectric fatigue testing machines and devices. Int. J. Fatigue.

[9] Ohnistova P., Piska M., Petrenec M., Dluhos J., Hornikova J., Sandera P. (2019). Fatigue life of 7475-T7351 aluminum after local severe plastic deformation caused by machining. Materials.

[10] Grover H.J., Gordon S.A., Jackson L.R. (1954). The Fatigue of Metals and Structures.

[11] Weibull W. (1939). A Statistical Theory of the Strength of Materials.

[12] Kugnel R. (1963). A relation between Theoretical Stress Concentration Factor and Fatigue Notch Factor Deduced from the Concept of Highly Stressed volume. J. Soc. Mater. Sci..

[13] Weibull W. (1951). A statistical distribution function of wide applicability. J. Appl. Mech..

[14] Esin A. (1980). A method for correlating different types of fatigue curve. Int. J. Fatigue.

[15] Philipp H.A. (1942). Einfluß von Querschnittsgröße und Querschnittsform. Forsch. auf dem Gebiete des Ingenieurwesens.

[16] Manson S.S., Muralidharan U. (1987). Fatigue life prediction in bending from axial fatigue information. Fatigue Fract. Eng. Mater. Struct..

[17] Özdeş H., Tiryakioğlu M., Eason P.D. (2017). On estimating axial high cycle fatigue behavior by rotating beam fatigue testing: Application to A356 aluminum alloy castings. Mater. Sci. Eng. A.

[18] Kurek A., Koziarska J., Kluger K. (2017). Łagoda fatigue life of 2017a-t4 aluminium alloy under different types. J. Mach. Constr. Maint..

[19] ISO-12107 (2012). Metallic Materials—Fatigue Testing—Statistical Planning and Analysis of Data.

[20] Sun C., Song Q. (2018). A method for predicting the effects of specimen geometry and loading condition on fatigue strength. Metals.

[21] Gaenser H.P. (2008). Some notes on gradient, volumetric and weakest link concepts in fatigue. Comput. Mater. Sci..

[22] Van Hooreweder B., Sas P., Boonen R., Moens D., De Coninck F. Experimental investigation of scaling laws for mechanical fatigue behaviour. Proceedings of the 8th National Congress on Theoretical and Applied Mechanics.

[23] Topoliński T., Cichański A., Mazurkiewicz A., Nowicki K. (2012). The relationship between trabecular bone structure modeling methods and the elastic modulus as calculated by FEM. Sci. World J..

[24] PN-EN ISO 6892-1:2016 (2016). Metallic Materials—Tensile Testing—Part 1: Method of Test at Room Temperature.

[25] ISO-1099 (2006). Metallic Materials—Fatigue Testing—Axial Force-Controlled Method.

[26] ISO-1143 (2010). Metallic Materials—Rotating Bar Bending Fatigue Testing.

[27] Rinne H. (2008). The Weibull Distribution.

[28] Ling J., Pan J. (1997). An engineering method for reliability analyses of mechanical structures for long fatigue lives. Reliab. Eng. Syst. Saf..

[29] Górecki T. (2011). Basics of Statistics with Examples in R.

[30] Nowak R.J. (1999). Mathematical Statistics.

[31] Ricci V. (2005). Fitting Distributions with R.

[32] R Core Team (2015). R: A Language and Environment for Statistical Computing.

[33] Howard B., Boyer H.E. (2003). Atlas of Fatigue Curves.

[34] Stephens R.I., Fatemi A., Stephens R.R., Fuchs H.O. (2001). Metal Fatigue in Engineering.

[35] Lee Y.L., Barkey M., Hathaway R., Pan J. (2005). Fatigue Testing and Analysis—Theory and Practice.

[36] Flaceliere L., Morel F. (2004). Probabilistic approach in high-cycle multiaxial fatigue: Volume and surface effects. Fatigue Fract. Eng. Mater. Struct..

[37] Muniz-Calvente M., de Jesus A.M.P., Correia J.A.F.O., Fernández-Canteli A. (2017). A methodology for probabilistic prediction of fatigue crack initiation taking into account the scale effect. Eng. Fract. Mech..

[38] Li Y., Song Q., Feng S., Sun C. (2018). Effects of loading frequency and specimen geometry on high cycle and very high cycle fatigue life of a high strength titanium alloy. Materials.

[39] ASTM E-739-91 (2006). Standard Practice for Statistical Analysis of Linear or Linearized Stress-Life (S-N) and Strain-Life (ε-N) Fatigue Data.

